# Yeast-based assays for the functional characterization of cancer-associated variants of human DNA repair genes

**DOI:** 10.15698/mic2020.07.721

**Published:** 2020-05-18

**Authors:** Tiziana Cervelli, Samuele Lodovichi, Francesca Bellè, Alvaro Galli

**Affiliations:** 1 Yeast Genetics and Genomics Group, Laboratory of Functional Genetics and Genomics, Institute of Clinical Physiology CNR, Via Moruzzi 1, 56125 Pisa, Italy.

**Keywords:** Saccharomyces cerevisiae, human DNA repair genes, cancer-associated variants, functional assays, humanized yeast strains

## Abstract

Technological advances are continuously revealing new genetic variants that are often difficult to interpret. As one of the most genetically tractable model organisms, yeast can have a central role in determining the consequences of human genetic variation. DNA repair gene mutations are associated with many types of cancers, therefore the evaluation of the functional impact of these mutations is crucial for risk assessment and for determining therapeutic strategies. Owing to the evolutionary conservation of DNA repair pathways between human cells and the yeast *Saccharomyces cerevisiae*, several functional assays have been developed. Here, we describe assays for variants of human genes belonging to the major DNA repair pathways divided in functional assays for human genes with yeast orthologues and human genes lacking a yeast orthologue. Human genes with orthologues can be studied by introducing the correspondent human mutations directly in the yeast gene or expressing the human gene carrying the mutations; while the only possible approach for human genes without a yeast orthologue is the heterologous expression. The common principle of these approaches is that the mutated gene determines a phenotypic alteration that can vary according to the gene studied and the domain of the protein. Here, we show how the versatility of yeast can help in classifying cancer-associated variants.

## DNA REPAIR AND CANCER

Genomic instability is an enabling trait for cancer development. The main factor determining genome instability is the alteration of the DNA damage response (DDR) [1]. DDR has a pivotal role in the protection of DNA from endogenous and environmental damage. DDR is composed of proteins sensing DNA damage, proteins responsible for the activation of checkpoints allowing the cells to repair DNA before entering S phase, and proteins actively involved in DNA repair. To cope with the different types of DNA damage, cells are equipped with several specialized DNA repair pathways: Base Excision Repair (BER), Mismatch Repair (MMR), Nucleotide Excision Repair (NER), Homologous Recombination (HR) and Non-Homologous End Joining (NHEJ) [2, 3]. BER is responsible for sensing and repairing DNA single-strand breaks (SSB). MMR is involved in repairing unsuitable insertions, deletions, and single nucleotide mismatched incorporation. The NER pathway corrects DNA adducts or UV-dimers induced by ultraviolet radiations. HR and NHEJ pathways play a major role in processing and repairing DNA double strand breaks (DSB) [4]. Moreover, during DNA replication, when the replication fork encounters a lesion in the DNA template and it is stalled, the post-replication repair (PRR) pathway allows to bypass the DNA damage and consequently to complete DNA replication [5].

Mutations in DNA repair genes may impact on cancer differently: they may predispose to cancer (cancer susceptibility genes), affect tumor progression or drug sensitivity of cancer cells. The present review is focused on DNA repair genes involved in cancer for which a functional yeast-based assay has been developed.

### Cancer predisposing mutations in DNA repair genes

Cancer susceptibility genes are those identified as mutated in the germline, usually in heterozygosis. The best characterized familial cancers associated with DNA repair genes are Lynch syndrome (or hereditary nonpolyposis colorectal cancers, HNPCC) and breast and ovarian cancer syndrome (HBOC). HNPCC is caused by inactivating mutations in MMR genes such as *MSH2, MSH6, MLH1, PMS1* and *PMS2* while HBOC is caused by inactivating mutations in genes involved in HR such as *BRCA1, BRCA2* [6, 7]. However, the advent of next generation sequencing (NGS) has allowed the identification of many germline polymorphisms in other genes playing a role in DNA repair that can be considered predisposing to cancer. HR genes such as *RAD51, RAD52,* promoting DNA exchange and stimulating homologous pairing, respectively, have been reported to predispose to HBOC. Similarly, pathogenic variants of the NHEJ genes *MRE11A* and *RAD50* may contribute to the risk of familial breast cancer [8]. Genetic polymorphisms in the NHEJ gene *XRCC6/KU70* are associated with several kinds of cancers; however, several contrasting results are reported and the effect of *KU70* polymorphisms on cancer risk is still ambiguous [9].

Polymorphisms of the BER gene *XRCC1* has been shown to be associated with colorectal, breast and ovarian cancer, prostate cancer, head and neck squamous cell carcinoma, pancreatic cancer [3]. Similarly, polymorphisms in NER genes have been identified in cancer types including lung and bladder [10]. The human homologue of the yeast gene *RAD3* named *XPD/ERCC2* which encodes for a DNA helicase involved in NER [11, 12], has been demonstrated to be associated with several cancers [13].

Mutations in at least one of the MMR genes *MSH2, MSH6, MLH1* and *PMS1* have been found to be associated not only with HNPCC, but also with other cancer types such as breast, bladder and gastric cancer [14–17].

Fidelity of DNA replication before cell division is fundamental for genome integrity and for preventing tumor development [18]. The fidelity of DNA replication is mainly due to the proofreading activity of DNA polymerase δ (Polδ) and ε (Polε), respectively Pol3 and Pol2 in yeast, that represent the principal polymerases involved in DNA replication. Besides their major role in DNA replication, Polδ and Polε participate in BER, NER, MMR and DNA DSB repair [19]. Despite the great advances in DNA sequence technology, the association between DNA polymerase defects or increased mutation and cancer was established rather recently [18, 20]. Mutations in *POLD1* and *POLE* genes encoding the catalytic subunit of Polδ and Polε, respectively, have been found in several types of cancer such as colorectal, endometrial, gastric and pancreatic cancer [18].

### Dysregulation of DNA repair genes and drug response

During the transformation process, somatic mutations, epigenetic silencing and dysregulation of DNA repair genes can occur. These alterations can render the cells either more vulnerable or more resistant to DNA damaging cancer drugs. For instance, upregulation of DNA repair genes can determine resistance to DNA damaging treatments such as radiotherapy and some chemotherapeutic agents. One example of frequently up-regulated DNA repair gene is the HR gene *RAD51*. Its overexpression has been observed in leukemia, breast, and pancreatic cancers [21]. *RAD18*, an E3 ubiquitin-linked enzyme, has a role in maintaining genome stability through multiple DNA repair pathways, including HR and PPR [22–24]. Several studies have shown that high expression of *RAD18* confers resistance to chemotherapy or radiotherapy in multiple human cancers [25–27]. Thus, targeted inhibition of DNA repair proteins could affect drug response and improve therapy efficacy [28, 29]. Moreover, when a DNA repair pathway is downregulated, cancer cells can become dependent on an alternative pathway to repair DNA damage [30]. This could represent an advantage in terms of therapeutic efficacy, because it allows exploitation of the principle of synthetic lethality. Cancer cells depleted of both pathways would be unable to repair DNA damage, therefore treatment with inhibitors of the alternative pathway could be lethal for cancer cells [30].

Importantly, the HR gene *RAD52* has been found to be synthetically lethal to the tumor suppressor gene *BRCA2*; this represents an important finding to design more precise cancer therapies [31, 32].

## YEAST-BASED FUNCTIONAL ASSAYS ON DNA REPAIR HUMAN GENES

NGS technology is making the sequencing of human genes very common in clinical genetics; this allows the identification of a large number of disease-associated mutations and thousands of polymorphisms in the human population [33]. Currently, the interpretation and assessment of the functional impact of disease-associated variants is a critical challenge [34, 35]. Genetic methods are often not informative due to the low frequency of the variant, which is, consequently, listed as “variant of unknown significance” (VUS). In hereditary cancer syndromes, the classification of the missense variants is urgently needed for risk assessment and to set up more precise therapies [36, 37]. One strategy to improve our knowledge on the functional impact of VUS is the use of functional assays. The purpose of functional assays is, not only, to classify and identify missense variants by assessing their impact on protein function, but also to correlate the biological function of the protein with its potential tumorigenic activity [38, 39]. Moreover, direct assessment of the variants by functional assays using simple genetic systems can help in speeding up the evaluation of newly identified cancer-associated variants [38, 40–42]. Several assays have been developed in yeast and mammalian cell lines to evaluate the functional impact of cancer-related mutations of DNA repair genes; this review describes different kinds of yeast-based functional assays and discusses potential clinical applications.

**Figure 1:**
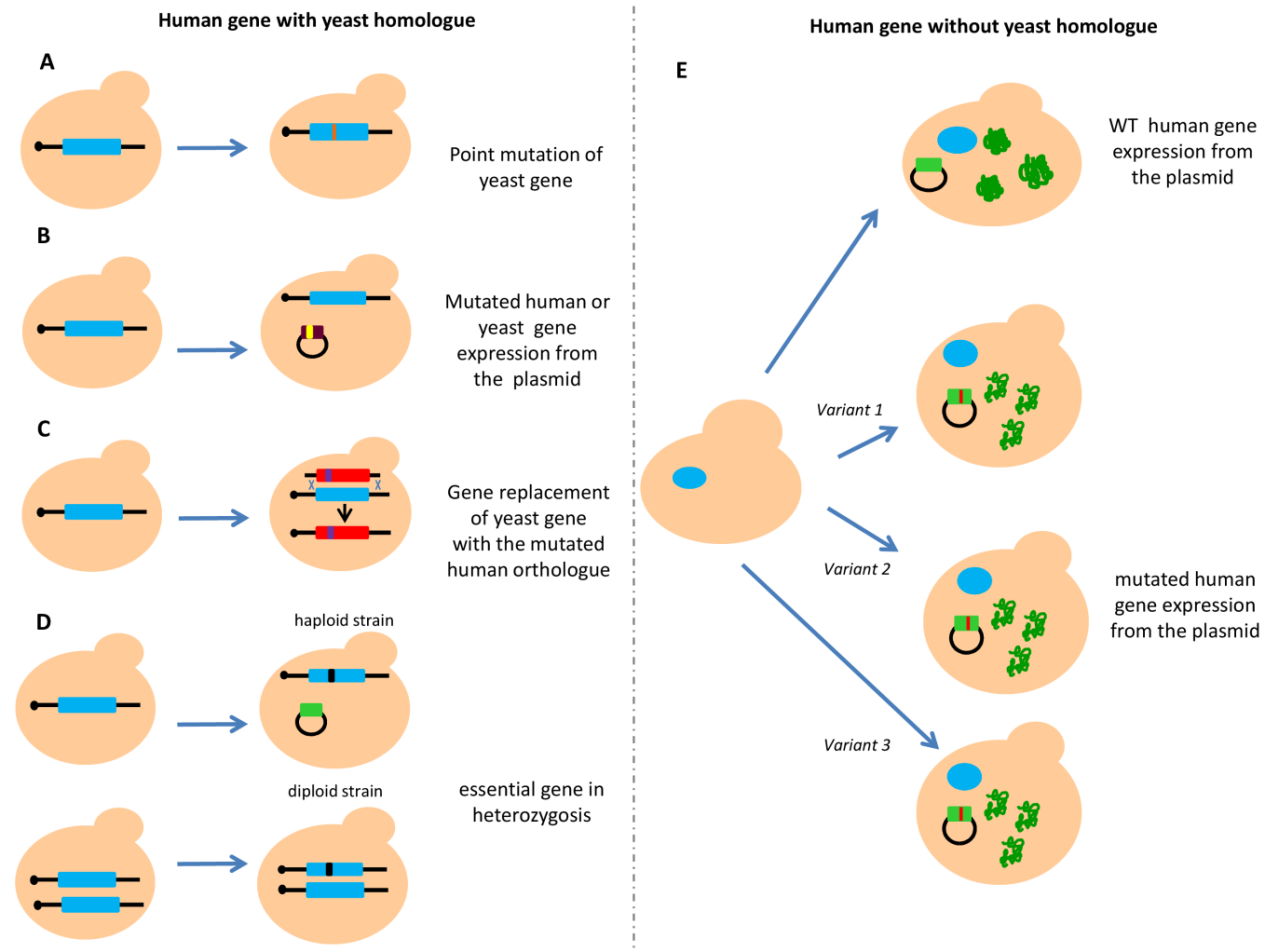
FIGURE 1: Constructions of humanized *S. cerevisiae* strains for functional analysis. Homology and complementation between yeast and human genes have to be considered for developing reliable functional assays. **(A)** When a yeast orthologue exists, yeast strains can be manipulated by directly inserting the mutation in the nucleotide corresponding to the human gene. **(B)** Mutants of human or yeast orthologous genes can be expressed from a plasmid. **(C)** Mutants of human or yeast orthologous genes can be replaced with the human counterpart. **(D)** In the case of an essential gene, functional analysis has to be evaluated in heterozygosis expressing the wild type form of the gene from a plasmid and mutating the endogenous one, or mutating one of the two alleles in a diploid strain. **(E)** When no homology between the human and yeast gene has been identified, the human gene is expressed from a plasmid.

Humans and the budding yeast *Saccharomyces cerevisiae* share thousands of protein-coding genes although they diverge by a billion years [43]. Interestingly, in several cases, the human gene can complement the deleted yeast gene [44]. Several reasons support the use of yeast as a model organism in cancer research. Yeast has contributed to the understanding of molecular mechanisms underlying cancer development through the discovery of crucial biological processes. Moreover, working with yeast is more economic compared to working with cell lines or animal models. Furthermore, construction of gene deletion strains, tagging proteins and site-specific mutagenesis are easier and faster than in human cells. Therefore, construction of “humanized” yeast, meaning a yeast strain expressing human genes or carrying mutation in an endogenous gene homologous to the human one, can be helpful in evaluating the functional consequences of human genetic variants found in several diseases [45–48]. When the yeast counterpart exists, functional assays can be developed by directly mutating the genomic copy of the yeast homologous gene (**Figure 1A**). Alternatively, the human gene or its orthologue can also be expressed from a plasmid (**Figure 1B**). Another approach implies the substitution of the yeast genomic copy with the corresponding human gene (**Figure 1C**). In the case of essential genes, the mutated gene has to be in heterozygosis; in haploid strains the wild type (WT) copy of the yeast gene is expressed from an episomal plasmid, in diploid strains, only one copy of the gene is mutated (**Figure 1D**). When the yeast counterpart does not exist, the human gene is usually expressed from a centromeric or multicopy plasmid carrying the human copy under the control of a yeast constitutive or inducible promoter (**Figure 1E**).

Yeast functional assays are mainly based on direct comparison of the phenotype conferred by the mutated protein with that of the WT one. These assays could be more reliable if the phenotype assessed is consistent to the biological function(s) of the protein. To determine the functional impact of human DNA repair gene variants, currently available yeast assays are based on the evaluation of forward and reverse mutation, DNA damage sensitivity, transcriptional activity, growth defect, protein mis-localization, intra- and inter-chromosomal recombination (**Table1**). As shown in **Table 1**, assays for characterizing human DNA repair proteins belonging to any pathway have been developed in yeast.

**TABLE 1:** DNA repair genes assayed in *Saccharomyces cerevisiae*.

**Pathway**	**Human gene**	**Yeast gene**	**Functional Assay**
MMR	*MSH2*	*MSH2*	Forward mutation [52], Reverse mutation [60]
	*MLH1*	*MLH1*	Forward mutation [51, 57, 59], Reverse mutation[56]
HR, PRR	*RAD18*	*RAD18*	UV, HU and MMS sensitivity [50]
HR	*RAD51D*	*RAD51*	MMS sensitivity [49]
	*RAD52*	*RAD52*	MMS sensitivity [49]
	*BRCA1*	*//*	TA [67, 70–72], SCP [63, 65, 75], YLP [64, 75], Liquid medium assay [75], Intra- and Inter-chromosomal HR assay [65], GR assay [76, 77]
	*BRCA2*	*//*	Intra- and Inter-chromosomal HR assay [66]
NHEJ	*KU70/XRCC6*	*YKU70*	UV, HU and MMS sensitivity [50]
NER	*XPD/ERCC2*	*RAD3*	UV, HU and MMS sensitivity [50]
MMR, NER, BER, HR, NHEJ	*POLE*	*POL2*	Forward mutation [20]
	*POLD1*	*POL3*	Forward mutation [61]

Several yeast-based assays have been developed to characterize human genes involved in Mismatch Repair (MMR), Homologous Recombination (HR), Post Replication Repair (PRR), Base and Nucleotide Excision Repair (BER, NER), and Non-Homologous End Joining (NHEJ). The name of the yeast and human gene is shown; *BRCA1* and *BRCA2* have no yeast homologue. TA, transcription activation assay; SCP, Small Colony Phenotype; YLP, Yeast Localization Phenotype; GR, gene reversion; MMS, methyl methane-sulfonate, HU hydroxyurea.

### Strategies to study DNA repair genes conserved in yeast and humans

The most attractive way to study the functional impact of cancer-related missense variants in human genes with an orthologue in yeast is to mutate native yeast genes to match the human sequences at the corresponding positions. This can be performed by aligning amino acid sequences of yeast and human proteins. To humanize specific positions within yeast genes, human and yeast genes have to share functional homology. Recently, we proposed a web tool that simultaneously finds the yeast homologous gene, identifies the corresponding variant(s) and determines the transferability of a human variant to the yeast counterpart by assigning a reliability score (RS) that may give helpful indications for potential accuracy of a functional assay to be developed [48]. The RS is assigned by an algorithm that computes functional data, type of mutation, amino acid conservation and chemistry of amino acid substitution. Mutations giving a positive RS are highly transferable to yeast and, therefore, yeast functional assays will be more reliable [48].

To our knowledge, study of the functional impact of mutated alleles that are localized in the native genome locus would be preferable to plasmid-based assays since gene expression is under the control of its natural promoter. This strategy has been exploited to study several DNA repair genes.

**Figure 2:**
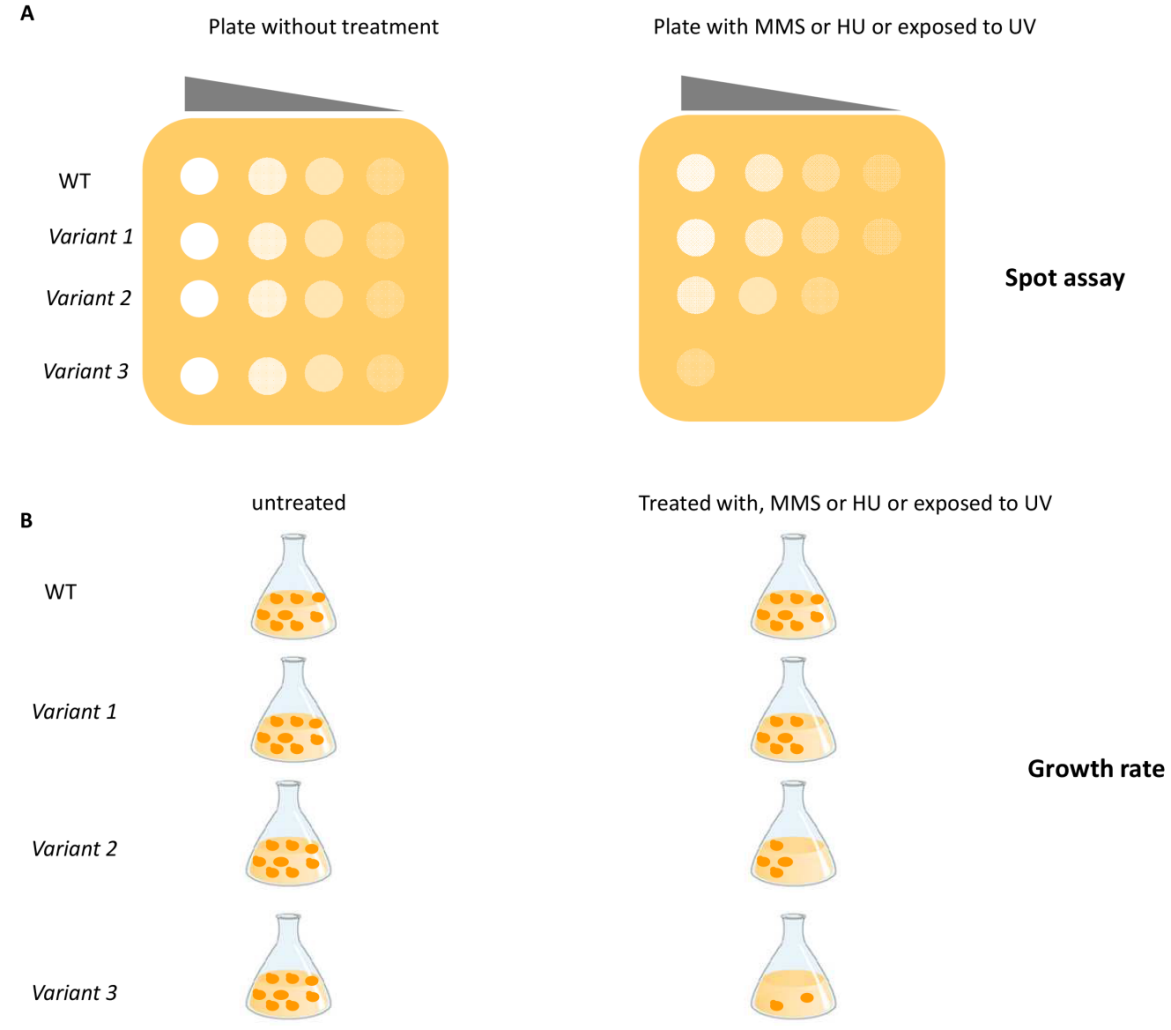
FIGURE 2: Schematic representation of functional assays based on DNA damage sensitivity. **(A)** Sensitivity to chemical or physical agents is determined by the spot assay. An overnight culture of yeast cells is serially diluted (10-fold dilution) and spotted onto plates containing the specific DNA damaging agent or are treated with UV. The effect of variants is determined by comparing growth of yeast expressing the variants vs WT. **(B)** The sensitivity to DNA damaging agents is evaluated by OD_600_ measurement of cell growth in liquid medium. Yeast cells are grown in medium containing the chemical agents or exposed to UV irradiation. The sensitivity of yeast cells carrying the gene variants is compared to yeast cells carrying the WT gene. MMS: methyl methane sulfonate; HU: hydroxyurea.

As the Rad51 and Rad52 protein sequences are highly conserved in human and *S*. *cerevisiae,* the functional impact of rare cancer-associated missense variants in both HR repair genes was evaluated by constructing yeast strains carrying correspondent mutated alleles (**Figure 1A**)[49]. A total of five predicted pathogenic variants, three located in the *RAD51* and two in the *RAD52* gene were analyzed in this study. Functional impact of the variants was evaluated by testing the sensitivity to DNA damaging agents and their effects on HR-mediated DSB repair (**Figure 2A**); three out of five variants studied conferred a hypersensitivity to methyl methane sulfonate (MMS) and defects in HR [49]. The effect of variants on HR has been studied by evaluating recombination intermediates and recombinants by one-dimensional (1D) and two-dimensional (2D) gel electrophoresis of the *HIS4LEU2* locus. This elegant approach has the limitation that it cannot be used for large scale analysis because its interpretation requires highly specialized expertise.

Recently, several yeast strains carrying single nucleotide substitutions in *RAD3, RAD18* and *YKU70* corresponding to mutated alleles of the human *ERCC2, RAD18* and *XRCC6* genes were constructed (**Figure 1A**) [50]. The functional impact of seven pathogenic variants (three located in the *RAD3* gene, one in *RAD18* and three in the *YKU70* gene) was evaluated by determining the sensitivity to DNA damaging agents such as UV, MMS and hydroxyurea (HU) by spot assay and by measuring growth rate (**Figure 2A** and **2B**); two variants showed a functional defect [50]. This study represents a good example on how yeast assays can consolidate results obtained with prediction tools (e.g SIFT: https://sift.bii.a-star.edu.sg/ and PolyPhen-2: http://genetics.bwh.harvard.edu/pph2/), even if the number of variants analyzed is too few to be applied to genetic oncology.

**Figure 3:**
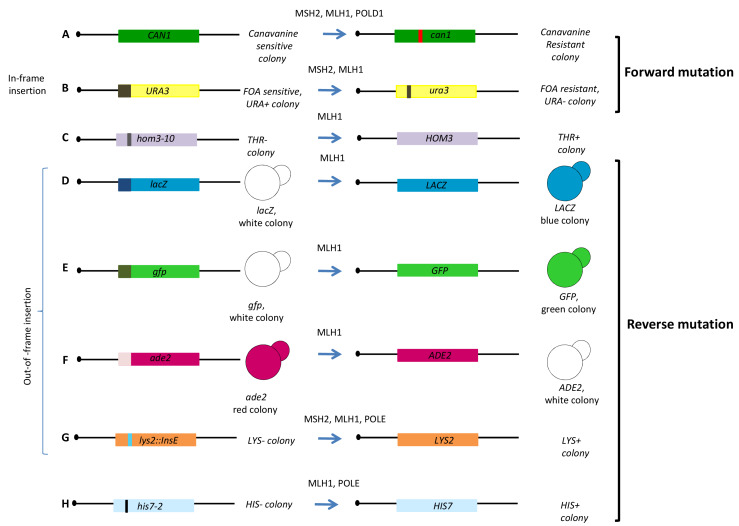
FIGURE 3: Schematic representation of the yeast functional assays for MMR genes. **Forward mutation** frequency/rate is evaluated by scoring the number of colonies becoming resistant to **(A)** Canavanine (*CAN1* to *can1*) or **(B)** 5-fluoroorotic acid (5-FOA, *URA3* to *ura3*). **Reverse mutation** is assessed by counting the number of colonies becoming able to grow in selective medium lacking threonine **(C)** (THR+; *hom3-10* to *HOM3*), **(D)** colonies becoming blue (*lacZ* to *LACZ*), **(E)** colonies becoming green (*gfp* to *GFP*), **(F)** colonies becoming white in medium lacking adenine (ADE+; *ade2* to *ADE2*), **(G)** colonies becoming able to grow in medium lacking lysine (LYS+; *lys2* to *LYS2*) and **(H)** colonies becoming able to grow in medium lacking histidine (HIS+, *hys7-2* to *HIS7*). The *URA3* gene used for forward reversion (B) contains an in-frame insertion of several nucleotides, therefore the gene is WT. Constructs of D, E, F and G contain out-of-frame insertion of several nucleotides, therefore the gene is mutated. Constructs of C and H contain a point mutation. Above the arrow are given the names of the human proteins studied with the assay.

Yeast strains carrying mutations corresponding to the homologous human gene or expressing the human variants have been exploited to evaluate the functional impact of variants located in the human MMR genes *hMLH1* and *hMSH2* (**Table 1**, **Figure 1B** and **1C**) [51–55]. One assay exploits the dominant-mutator effect that WT human MMR proteins have in MMR proficient yeast strain; in fact, the expression of *hMLH1* WT gene in yeast increases the spontaneous mutation rate measured by either a forward or reverse mutation assay. The mutator phenotype conferred to yeast by *hMLH1* WT is suppressed when a pathogenic variant is expressed [51]. By using a forward mutation assay at the *CAN1* locus (**Figure 3A**) and reverse mutation assays at the *hom3-10* (**Figure 3C**) locus and *lacZ* (**Figure 3D**), the authors demonstrated that 13 out of 27 *hMLH1* cancer-associated variants showed a functional impact. More recently, the dominant mutator effect has been evaluated for 101 *hMLH1* cancer-associated variants by three yeast forward mutations assays (**Figure 3D**, **3E** and **3F**). The authors classified the variants in four categories depending on the results of the *LacZ, GFP*, and *ADE2* assays. In this way, they could evaluate functionally subtle variants such as those responding only to one assay. A total of 70 variants gave loss of the mutator phenotype suggesting that this yeast assay could be a simple method to analyze a large number of variants [56].

Functional consequences of several cancer-associated *hMSH2* and *hMLH1* missense variants were also evaluated by mutating the corresponding yeast gene and determining the phenotype as compared to the WT strain. When a mutation has functional impact, it increases the spontaneous mutation rate conferring a mutator phenotype. In one study, twelve out of 17 *MLH1* yeast variants corresponding to *hMLH1* cancer-associated mutations, showed a strong mutator phenotype assessed by forward mutation at the *URA3* locus (**Figure 3B**) [53]. In another study, haploid yeast strains carrying six *MLH1* missense mutations that correspond to germline mutations found in human cancer patients, displayed a strong mutator phenotype when tested by forward (**Figure 3A**) and reverse mutation (**Figure 3G** and **3H**) [57]. A total of 28 alleles of yeast *MLH1* corresponding to non-truncating human mutant alleles were studied in a reversion assay (**Figure 3G**); 24 alleles were able to induce a significant increase in the reversion rate [58]. Interestingly, yeast strains expressing the human mutated *MLH1* alleles under control of the native yeast promoter, have been constructed in the MMR defective background (**Figure 1C**). The functional impact of eight *hMLH1* variants was determined by evaluating the effect on the mutation rate; three out of five pathogenic variants increased the mutation rate at the *URA3* locus (**Figure 3B**) suggesting that complementation of yeast MMR defect by human corresponding alleles could be proposed as a system to characterize cancer-associated genetic variants [59]. Functional assays have also been performed for the MMR gene *MSH2* [52, 53, 60]. As observed for *MLH1*, yeast *msh2*Δ shows a mutator phenotype that can be complemented by expressing *MSH2* WT from a plasmid. Exploiting the mutator phenotype of yeast *msh2*Δ*,* the effect of five yeast *MSH2* missense mutations analogous to those found in h*MSH2* were assayed by a reversion assay at the *LYS2* locus (**Figure 3G**) [60]. In another assay, 54 missense mutations were introduced in the cognate positions in yeast *MSH2* and tested for functional defects and assayed for their ability to induce forward mutations at the *URA3* and *CAN1* locus (**Figure 3A** and **3B**). 34 cancer-associated variants conferred a mutator phenotype [52].

The assessment of the functional impact of cancer-associated DNA polymerase δ and ε variants (*POLD1* and *POLE*) has been evaluated in yeast by constructing strains carrying the correspondent mutated amino acid identified by aligning yeast and human sequences. Since the yeast counterparts *POL3* and *POL2*, respectively, are essential in yeast, functional assays have to be set up in diploid strains (with mutations in heterozygosis) or in haploid strains expressing WT *POL* genes from a plasmid (**Figure 1D**) [20, 61]. The assays are based on the mutator effects of the cancer-associated Pol variants by comparing forward and reverse mutation rates observed in yeast strains carrying the variants with those observed in strains carrying the WT gene (**Table1**, **Figure 3A**, **3G** and **3H**). A total of 19 cancer-associated DNA polymerase variants were analyzed in yeast, 13 for Polε, and six for Polδ [20, 61]. Cumulatively, these results showed that eight Polε and 1 Polδ variants confer a mutator phenotype. Moreover, one Polδ variant (Polδ-R696W which is analogous to the human Polδ-R689W variant) and one Polε (Pol2-R252H which is analogous to Polε-R231H) increased the mutation rate of the MMR defective yeast strain, suggesting a functional interplay between replication fidelity and MMR [20, 61, 62].

### Strategies to study DNA repair human genes lacking yeast orthologues

**Figure 4:**
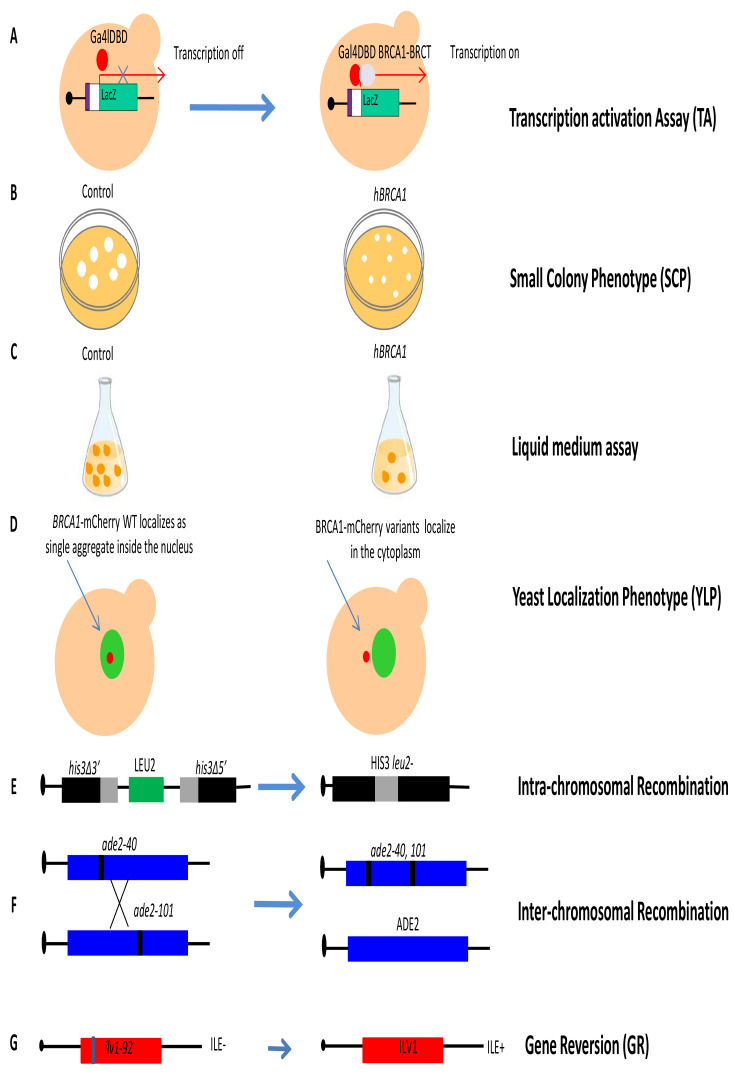
FIGURE 4: Schematic representation of the yeast functional assays for *BRCA1/2* (A) Transcription activation assay (TA): The BRCT domain of BRCA1 is cloned in frame with the GAL4 or LexA DNA binding domain (DBD). WT BRCT fused to DBD is able to activate transcription of the reporter gene LacZ under control of the minimal promoter (white) containing the binding sequences (violet) recognized by the GAL4 or LexA DBD. Variants affecting BRCT domain activity are not able to activate LacZ transcription. **(B) Small colony phenotype assay (SCP)**: yeast cells expressing *BRCA1* (from pADH1 fused to the activation domain of GAL4, or from inducible pGAL1) form colonies considerably smaller than controls after incubation at 30°C. Yeast colonies re resuspended in water, and the number of cells per colony are determined by counting. Small colonies correlate with slow growth, therefore the inhibition of growth determined by *BRCA1* expression can be performed also in liquid medium. **(C) Liquid Medium assay**: this assay monitors the growth defect of yeast cells expressing *BRCA1* as in the SCP, but in liquid instead of solid medium. **(D) Yeast localization phenotype assay (YLP)**: this assay is performed in yeast cells expressing *BRCA1* cloned in frame with m-Cherry at C-terminus (*BRCA1*-mCherry) under control of the inducible GAL promoter. Yeast cells expressing WT or *BRCA1* variants fused to mCherry are induced for 4 h with galactose before live fluorescent microscopy analyses. The nucleus is identified by the expression of the nuclear protein Nup133 fused to GFP (Nup133-GFP, green). Whereas the WT *BRCA1* protein shows mainly nuclear localization (Red spot), pathogenic variants show prevalent cytoplasmic localization. **(E) Intra-chromosomal recombination**: the yeast strain carries the two *his3* alleles separated by the *LEU2* marker and by the plasmid DNA sequence, one with a deletion at the 3' end and the other with a deletion at the 5' end, which share 400 bp of homology (gray box). An intra-chromosomal recombination event leads to *HIS3* reversion and loss of *LEU2* determined by counting colonies grown in medium lacking histidine (HIS3+). **(F) Inter-chromosomal recombination**: the yeast strain contains the two alleles *ade2-40* and *ade2-101*, located in two homologous chromosomes. An inter-chromosomal recombination event leads to WT *ADE2* determined directly by counting colonies grown in medium lacking adenine (ADE2+). **(G) Gene reversion (GR)**: the yeast strain carries the *ilv1-92* that allows the assessment of gene reversion to *ILV1* by direct counting colonies grown in medium lacking isoleucine (ILE+).

Functional assays of human DNA repair genes lacking yeast orthologues have been performed for *BRCA1* and *BRCA2* (**Table1**). In order to develop assays for functional characterization of cancer-associated variants located in *BRCA1* and *BRCA2*, yeast strains overexpressing full length *BRCA1* or *BRCA2* under the control of a constitutive (pADH) or inducible promoter (pGAL1) have been constructed (**Figure 1D**) [63–67]. The *BRCA1* gene encodes a nuclear phosphoprotein that plays a role in DNA damage repair, transcriptional regulation, cell cycle control and ubiquitylation. The BRCA1 protein exhibits several functions, including ubiquitin ligase activity, as well as nucleic acid binding activity and transcription coactivator activity. The protein consists of three main domains, the N-terminus -RING motif, the internal serine containing domain (SCD) and the BRCA1 C-terminus (BRCT) [68, 69]. The BRCT domain of BRCA1 has been found to act as transactivation domain, therefore to study the impact of mutations of this domain, a transcription activation assay (TA) has been proposed (**Figure 4A**) [70]. This assay is performed using a chimeric protein consisting of the C-terminus of BRCA1 (1396aa – 1863aa), which includes the BRCT domain, fused to the Gal4 or LexA DNA binding domain (DBD). This protein is able to activate transcription of the reporter gene *LacZ* integrated in the yeast genome or carried on an episomal plasmid, regulated by a minimal promoter containing Gal4 or LexA binding sites. Quantification of the reporter gene product permits an indirect assessment of the transcriptional activity mediated by the BRCA1 fusion protein and comparison between WT and variants allows to evaluate the impact of mutations [70, 71]. Carvalho *et al.* analyzed 24 variants located in the BRCT-BRCA1 domain and showed that the TA assay correctly classified all 24 variants [71]. More recently, Fernandes *et al.* classified 102 (99 missense and three truncating) variants located in the BRCA1-BRCT domain, by integrating data from a yeast-based TA assay with other functional data from a mammalian TA assay [72]. Overall, yeast the TA assay displayed 100% specificity and sensitivity [73]. These results indicate that the yeast TA assay is accurate and can be very helpful to classify novel BRCA1 variants.

The expression of human *BRCA1* WT in the budding yeast *S. cerevisiae* was found to strongly inhibit growth on solid medium (**Figure 4B**) [74]. This peculiar phenotype has been exploited to develop a simple functional assay named small colony phenotype assay (SCP) based on the ability conferred by *BRCA1* pathogenic variants expression to restore yeast growth. The evaluation of the functional effects is determined by counting directly the number of cells per colony (**Figure 4B**) [63]. In total, 28 missense mutations were introduced in the BRCA1-BRCT domain; eleven mutations showed increased yeast colony size as compared to yeast expressing *BRCA1* WT suggesting a disruption of BRCT structure and/or function [63]. In addition, our group performed SCP assays to evaluate the functional impact of four mutations located in the BRCA1-BRCT domain; basically, we confirmed that this assay could be helpful to classify BRCT variants [65]. More recently, this assay has been validated by investigating the effect of as many as 40 (25 pathogenic and 15 neutral) variants located in almost all BRCA1 protein domains [75]. In this study, the specificity and sensitivity of the SCP assay was found to be 93% and 96% [75]. The same authors also validated the liquid medium assay that evaluates the growth inhibitory effect of BRCA1 in liquid medium over 15 hours (**Figure 4C**); however, this assay has less specificity and sensitivity than the SCP assay [75].

In the yeast localization phenotype (YLP) assay, the expression of *BRCA1* WT fused to the mCherry red fluorescent protein provides qualitative information concerning the subcellular localization of BRCA1 since BRCA1-mCherry WT accumulates in a single inclusion body in the yeast nucleus while mutated BRCA1 pathogenic variants mainly localize in the cytoplasm (**Figure 4D**) [64]. This assay was validated by studying a total of 40 variants (25 pathogenic and 15 neutral) previously analyzed with the SCP assay; results indicated that the YLP assay has less sensitivity (84%) and the same specificity (93%) as SCP [73, 75].

Other assays to evaluate the impact of missense mutations in *BRCA1* have been developed by our group. Since *BRCA1* is involved in DNA repair, we developed two HR assays to evaluate the impact of variants on intra- and inter-chromosomal recombination (**Figure 4E** and **4F**), and one assay to assess their effect on gene reversion (GR) at the *ilv1-92* locus (**Figure 4G**) [65, 76, 77]. In our first explorative paper, we demonstrated the validity of the assay analyzing the effect of twelve *BRCA1* variants (four pathogenic, seven neutral and one not classified) in a diploid yeast strain carrying the two different HR substrates to measure intra-chromosomal and inter-chromosomal recombination events [65]. Results demonstrated that the four pathogenic variants significantly induced at least one HR event. The effect of BRCA1 pathogenic variants on GR was also evaluated in a haploid yeast strain. Results indicated that four out of five pathogenic variants and to out of six neutral variants induced a significant GR increase confirming the yeast system as a valuable tool to classify uncharacterized *BRCA1* variants [76, 77].

Intra-chromosomal and inter-chromosomal HR assays have also been used to evaluate the effect of missense variants of *BRCA2*. The expression of *BRCA2* WT in yeast increases both intra-chromosomal and inter-chromosomal HR [66]. Spugnesi *et al.* observed that one pathogenic variant did not affect HR, while the neutral variants significantly increased HR to the level of *BRCA2* WT [66]. To understand the potential application of this assay in clinical genetics, a larger number of classified variants needs to be studied.

## CONCLUSIONS AND PERSPECTIVES

Overall, the advantage of functional assays is to provide results that can be applied to clinical genetics in order to classify novel variants. For this purpose, functional assays need to be statistically validated using a significant number (at least 40) of classified variants (pathogenic and benign) and the WT protein as control [75]. Cancer-associated variants of *BRCA1* and the MMR genes *MSH2, MLH1* are the most studied in yeast and several functional assays have been developed in order to classify VUS. Functional assays to assess the impact of mutations of MMR genes are based on the evaluation of the effect of mutations on the mutator phenotype i.e. the pathogenic variants should affect mutation rate as compared to the WT. The International Society for Gastrointestinal Hereditary Tumours (InSiGHT, https://www.insight-group.org/) established criteria and guidelines for the classification of new MMR variants, and provided a list of MMR functional assays that could be helpful to classify new variants [78]. InSiGHT also indicates that yeast-based MMR functional assays should not be considered definitive for variant classification, but only to assess pathogenicity. This assumption is based on the detailed examination of results of yeast assays for variants considered benign: discordant results were reported for 8/19 (42%) variants assayed, as compared to only 1/18 (5.5%) in mammalian assays [78]. However, this conclusion is drawn from a very limited number of variants and requires a larger data set for a more accurate analysis. In addition, the difficulties experienced in interpreting apparently discordant data from functional assays emphasize the importance of assay validation and standardization.

A range of different assays have been used to assess the effects of *BRCA1* variants on protein function, some limited to measure the functional impact of variants within a specific domain (TA assay and SCP), and others to measure output relevant to variants located anywhere in the coding region (HR and GR assay) [38, 77]. The consortium ENIGMA (Evidence-based Network for the Interpretation of Germline Mutant Alleles, http://enigmaconsortium.org/) has developed *BRCA1/2* variant classification criteria that utilize statistical and qualitative methods including functional data to assess the clinical significance of variants. Particularly, ENIGMA provides guidance and rules on how to integrate *BRCA1/2* protein functional data for classification of *BRCA1/2* missense variants. To evaluate the strength of these assays as predictors of the clinical significance of newly identified VUS, sensitivity and specificity of assays should be determined using previously classified missense variants. For multi-domain proteins such as BRCA1, some assays need an integrative statistical validation by analyzing large numbers of variants and by comparing data from them with data from other analysis [79]. Yeast-based SCP, YLP and TA assays have been recently validated and therefore could be useful for classifying VUS according to ENIGMA rules [75] [72].

Functional assays have traditionally been applied to each newly identified VUS, but the rapid increase of VUS has prompted the proposal of novel strategies to determine clinical significance of thousands of variants simultaneously [80]. The approach named multiplexed assay of variant effect (MAVE) could really contribute to evaluate functional consequences of human genetic variation [81]. MAVE has allowed the assessment of thousand variants located in coding sequence, enhancers and promoters [82]. Importantly, MAVE analysis have been carried out in several genetic systems including yeast [83, 84]. In general, MAVE is based on the construction of a library of variants to be introduced in the cells, and on the evaluation of GFP-based recombination systems or growth assays [85]. Therefore, selection and read-out methods to assess the functional impact of variants are crucial for VUS classification. Recently, Findaly *et al.* analyzed the functional consequences of almost 4,000 *BRCA1* variants located in 13 exons by using a CRISP-Cas9 saturation mutagenesis approach. Functional scores (FS) are determined by measuring the effect of the BRCA1 variants on growth in a haploid cell line [86]; moreover, FS are highly accurate to predict pathogenicity with sensitivity and specificity over 95%. Very recently, a novel high-throughput method for functional analysis was developed to assess the pathogenicity of VUS within *BRCA2*; out of 244 total variants, as many as 186 pathogenic variants were identified. Sensitivity and specificity of this assay were estimated to be 95% [87].

MAVE strategy has been applied to several clinically relevant proteins including the tumor suppressor *TP53* and *PTEN* producing a large amount of data that could be potentially useful for clinical applications. Recently, detailed recommendations on how to perform MAVE data collection and interpretation, and even to design a MAVE screening, have been published [82]. Indeed, these new methods may provide FS that can be used to classify the variants; however, some of them do not rely on the biological function of the protein and a direct comparison to WT is lacking. Therefore, to investigate functional consequences of cancer-associated variants in validated yeast-based assays that rely on the biological function of the protein would be preferable, because data are easier to interpret and compare to other functional assays. Moreover, for a more complete evaluation of the functional impact of many variants, comparative studies using several assays are preferable, particularly for “intermediate” or “low risk” variants. In the next future, it would be very helpful to apply MAVE in yeast for functional evaluation of variants located in DNA repair genes such as *Rad51/52* or MMR genes; the challenge is to develop reporter systems that score for mutator or HR effects.

## Abbreviations:

BER: – base excision repair;
BRCT: – BRCA1 C-terminus;
DDR: – DNA damage repair;
DSB: – double strand break;
ENGIMA: – Evidence-based Network for the Interpretation of Germline Mutant Alleles;
FS: – functional score;
GR: – gene reversion;
HBOC: – breast and ovarian cancer syndrome;
HNPCC: – hereditary nonpolyposis colorectal cancer;
HR: – homologous recombination;
InSiGHT: – International Society for Gastrointestinal Hereditary Tumours;
MAVE: – multiplexed assay of variant effect;
MMR: – mismatch repair;
MMS: – methyl methane sulfonate;
NER: – nucleotide excision repair;
NGS: – next generation sequencing;
NHEJ: – non-homologous end joining;
Pol: – polymerase;
PRR: – post-replication repair;
RS: – reliability score;
SCP: – small colony phenotype;
TA: – transcription activation;
VUS: – variant of unknown significance;
WT: – wild type;
YLP: – yeast localization phenotype.
